# Electroporation of DNA into *Physarum polycephalum* Mitochondria: Effects on Transcription and RNA Editing in Isolated Organelles

**DOI:** 10.3390/genes7120128

**Published:** 2016-12-14

**Authors:** Jonatha M. Gott, Gregory M. Naegele, Scott J. Howell

**Affiliations:** 1 Center for RNA Molecular Biology, Case Western Reserve University, Cleveland, OH 44120, USA; gmnaegele@gmail.com; 2 Visual Sciences Research Center, Case Western Reserve University, Cleveland, OH 44120, USA; sjh36@case.edu

**Keywords:** RNA editing, *Physarum polycephalum*, mitochondria, electroporation

## Abstract

Mitochondrial RNAs in the acellular slime mold *Physarum polycephalum* contain nucleotides that are not encoded in the mitochondrial genes from which they are transcribed. These site-specific changes are quite extensive, comprising ~4% of the residues within mRNAs and ~2% of rRNAs and tRNAs. These “extra” nucleotides are added co-transcriptionally, but the means by which this is accomplished have not been elucidated. The *cox1* mRNA also contains four sites of C to U changes, which occur post-transcriptionally, most likely via targeted deamination. The currently available in vitro systems for studying *P. polycephalum* editing are limited in that the template is the entire ~63,000 bp mitochondrial genome. This presents a significant challenge when trying to define the signals that specify editing sites. In an attempt to overcome this issue, a method for introducing DNA into isolated *P. polycephalum* mitochondria via electroporation has been developed. Exogenous DNA is expressed, but the transcripts synthesized from these templates are not edited under the conditions tested. However, transcripts derived from the mitochondrial genome are accurately edited after electroporation, indicating that the editing machinery is still functional. These findings suggest that this method may ultimately provide a feasible approach to elucidating editing signals.

## 1. Introduction

Many species produce RNAs whose sequences differ from the DNA from which they are transcribed. The processes that result in these site-specific alterations are collectively referred to as RNA editing [1]. Editing mechanisms vary widely, resulting in changes at either the base or nucleotide level [2]. Mitochondrial RNAs are extensively edited in a number of organisms. Maturation of higher plant mitochondrial RNAs involves hundreds of C to U changes resulting from targeted post-transcriptional deamination reactions, while some lower plant mitochondrial RNAs contain thousands of C to U changes and hundreds of U to C changes [3]. Base changes of multiple types are also prevalent in mitochondrial RNAs in certain dinoflagellates [4,5], although the underlying mechanism remains unclear. Widespread changes at the nucleotide level occur in the mitochondria of kinetoplastid protozoa and myxomycetes. Kinetoplast RNAs are processed by a series of guide RNA-directed cleavage and ligation reactions, resulting in the insertion of thousands of added uridines and removal of hundreds of encoded Us [6], while over 1300 non-encoded nucleotides are added to mitochondrial RNAs in *Physarum polycephalum* [7]. 

At least three distinct RNA editing mechanisms are used during mitochondrial gene expression in *P. polycephalum* [8]. The majority of editing events in *P. polycephalum* mitochondria involve the precise insertion of non-encoded nucleotides (nt), ~95% (1255 of 1324 nt) of which are single C insertions. Other ribonucleotides are also added at a limited number of defined sites, either singly or in pairs. Rarer forms of editing include a deletion of three encoded A residues [9], the replacement of the 5′ nucleotide of two mitochondrial tRNAs [10], and four C to U changes [11]. In total, site-specific changes are present in 37 of the 39 mRNAs, all three rRNAs, and the five tRNAs encoded in the *P. polycephalum* mitochondrial genome. Editing is extremely accurate and highly efficient based on both characterization of transcripts from individual genes [9,10,11,12,13,14,15,16] and sequencing of the entire mitochondrial transcriptome [7].

The mechanism of nucleotide insertion in *P. polycephalum* mitochondria is distinct from the guide RNA-directed U insertion/deletion form of editing described in trypanosomes and the polymerase stuttering utilized in viral systems [17]. We are interested in elucidating the *cis*-acting elements and *trans*-acting factors involved in RNA sequence alterations within *P. polycephalum* mitochondria, and have established two in vitro systems in which to study editing, both of which rely on transcription/editing complexes formed in vivo [18,19]. Using these systems, we have demonstrated that insertion editing occurs co-transcriptionally, i.e., the extra nucleotides are added at the 3′ end of the nascent RNA during synthesis [17], and that the template elements required for the insertion of non-encoded nucleotides are limited to ~18 base pairs (bp) centered around the editing site [20]. However, in order to definitively identify the *cis*-elements that direct editing of *P. polycephalum* mitochondrial transcripts, it will be necessary to achieve transcription and editing from a template that can be easily manipulated in vitro. 

Electroporation has been used previously to introduce DNA into isolated mitochondria. An early study using mammalian mitochondria demonstrated DNA uptake and the effects of field strength on mitochondrial function, but did not examine transcriptional activity [21]. This methodology was subsequently adapted to study transcription, splicing, and RNA editing in mitochondria from wheat [22,23,24,25,26,27], maize and sorghum [28,29], and potato [30,31]. Accurate C to U editing of transcripts derived from chimeric genes was observed in each of these systems, allowing localization of the *cis*-acting signals essential for editing [22,25]. 

Here we describe the development of methods for the introduction of DNA into *P. polycephalum* mitochondria and provide evidence that exogenous DNA is expressed, albeit at low levels. In these initial studies, no evidence of either nucleotide insertion or C to U changes was observed in transcripts from the introduced templates. However, transcription from endogenous genes is not significantly affected by electroporation at moderate field strengths, and the transcripts synthesized from the mitochondrial genome after electroporation are still largely edited. Thus, the editing apparatus is not inactivated under these conditions, providing a starting point for future investigations of editing signals.

## 2. Materials and Methods

### 2.1. DNAs Used for Electroporation

**Figure 1:**
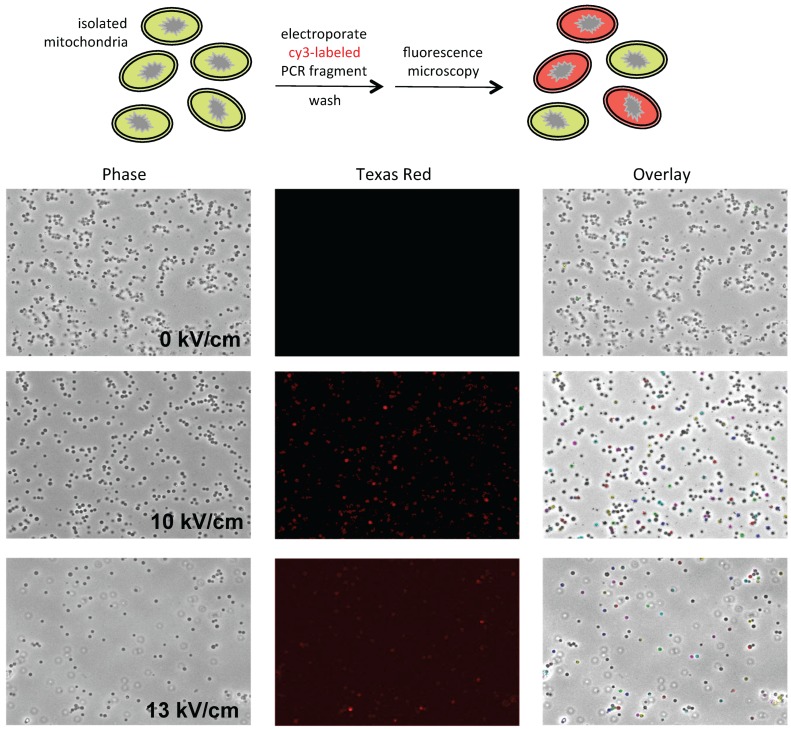
Uptake of cy3-labeled DNA into isolated mitochondria upon electroporation: (**Top**) Schematic representation of the experiment; and (**Bottom**) (**left**) phase contrast images of purified mitochondria incubated with a 847 bp cy3-labeled PCR fragment without (0 kV/cm) or with electroporation at either 10 kV/cm or 13 kV/cm; (**center**) visualization of cy3 fragment uptake via fluorescence microscopy; and (**right**) overlay of the fluorescent signals (shown in color) onto the respective phase images.

**Figure 2:**
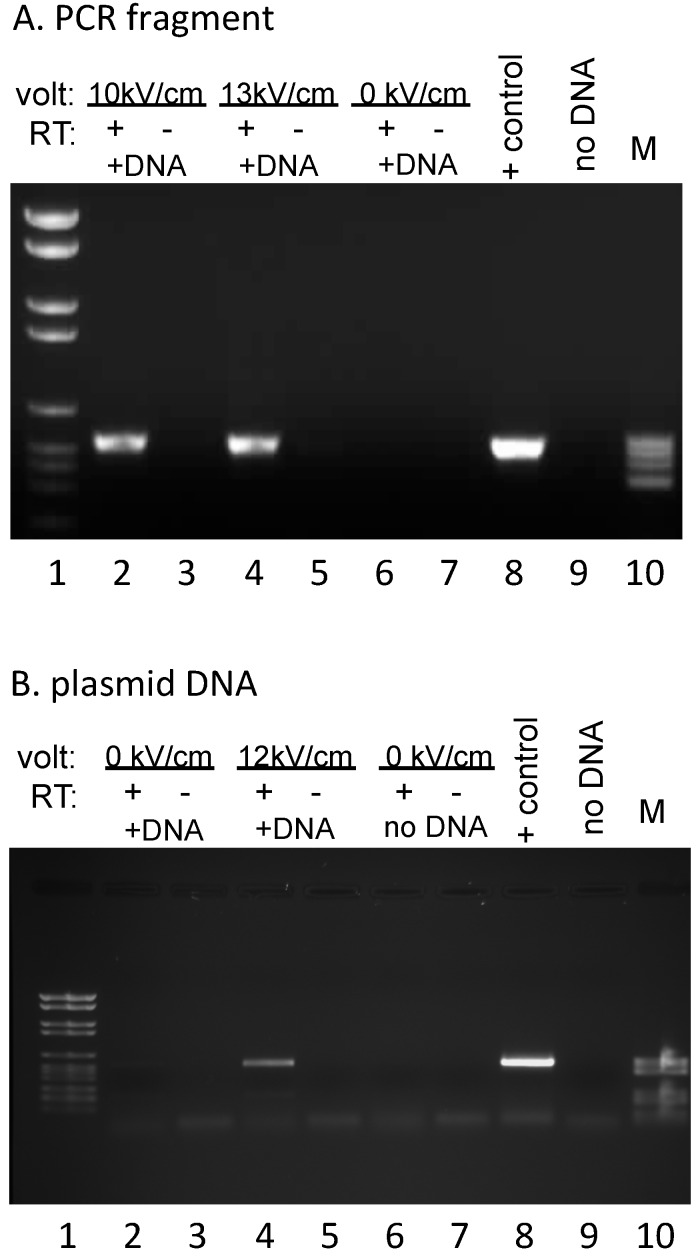
DNA introduced into mitochondria is transcribed: (**A**) Reverse transcription PCR (RT-PCR) products derived from RNA synthesized after incubation of isolated mitochondria with a 847 bp cy3-labeled PCR fragment with (10 kV/cm, 13 kV/cm) or without (0 kV/cm) electroporation. The +RT/−RT designation indicates reverse transcription reactions done in the presence or absence of enzyme, respectively. Lanes 1 and 10 contain size standards VI and V (Roche), respectively (M); PCR controls are shown in lanes 8 and 9. (**B**) RT-PCR products derived from RNA synthesized after incubation of isolated mitochondria with a 3.9 kb plasmid with (12 kV/cm) or without (0 kV/cm) electroporation. Samples in lanes 6 and 7 demonstrate the lack of background with total mitochondrial RNA (no exogenous DNA, no electroporation) with this set of plasmid-specific PCR primers. Other labels as in A.

**Figure 3:**
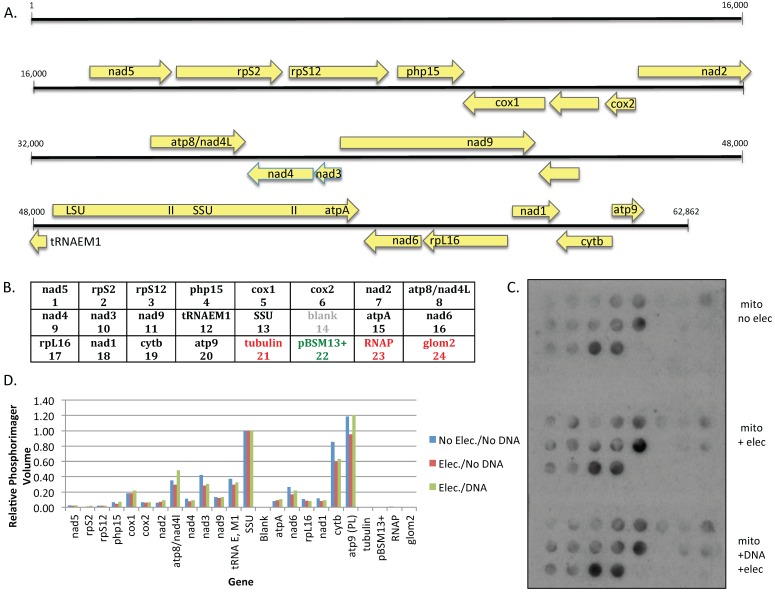
Transcription of endogenous genes is not altered by electroporation: (**A**) Transcript map of the genes probed in dot blot experiments using gene designations from [7]. (**B**) Grid positions of immobilized PCR fragments; (**C**) Dot blots probed with labeled RNA synthesized by: mitochondria before electroporation (**top**); mitochondria after electroporation in the absence of exogenous DNA (**middle**); and mitochondria after electroporation in the presence of exogenous DNA (**bottom**). (**D**) Quantification of the data in (**C**) showing relative expression levels of individual genes under each of the three conditions.

**Figure 4:**
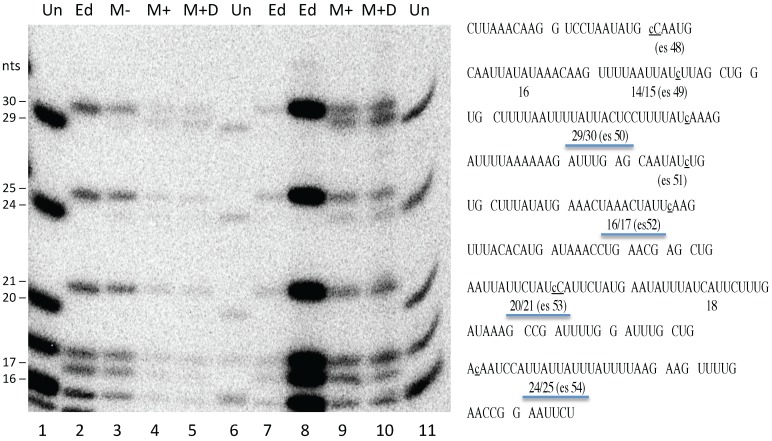
The editing machinery is still functional after electroporation. Gel electrophoresis of RNase T1 products generated from S1 nuclease-protected RNA fragments that encompass the last seven editing sites of the *atpA* mRNA. Internally labeled RNAs were synthesized by mitochondria before electroporation (M−, lane 3), mitochondria after electroporation in the absence of exogenous DNA (M+, lanes 4 and 9), and mitochondria after electroporation in the presence of exogenous DNA (M +D, lanes 5 and 10). Different amounts of the same RNase T1 digests were loaded in lanes 4 and 9 and lanes 5 and 10. Multiple dilutions of RNase T1 digests of S1 nuclease-protected unedited (Un, lanes 1, 6, and 11) and edited (Ed, lanes 2, 7, and 8) control transcripts were run alongside for comparison. The sequence of the RNase T1 fragments from the protected region of the *atpA* mRNA is shown at the right, with the C residue added at editing sites 48 through 54 (es48–es54) indicated by a lower case c. Easily resolvable RNase T1 fragments diagnostic of editing are underlined and the size in nucleotides (nts) of the edited and unedited fragments are indicated on the sequence and to the left of the gel.

All plasmids used in this study contained the promoter region that drives transcription of the mitochondrial ribosomal RNA genes (large and small subunit rRNAs and 5S rRNA). The plasmid (#809) used in the experiments in Figure 1, Figure 2B, and Figure S1 contains the entire 125 bp intergenic region upstream of the *P. polycephalum* mitochondrial large subunit rRNA (*LSU*) gene and 97 bp of the transcribed portion of the *LSU* gene fused via a linker to 445 bp derived from the *P. polycephalum* mitochondrial *cox1* gene and inserted into the HindIII and EcoRI sites of vector pBSM13+ (Agilent Technologies, Santa Clara, CA, USA). The linker sequence provides a unique primer binding site which, along with the 5 bp block mutation introduced near the 3′ end of the *cox1* portion (shown in Figure S1), ensures that reverse transcription PCR (RT-PCR) products derived from fusion gene transcripts can be distinguished from those arising from the endogenous *cox1* gene. The 847 bp PCR product used in the experiments shown in Figure 1 and Figure 2A contained the entire insert plus flanking sequences derived from the vector. The PCR product was purified using a QIAquick PCR purification kit (Qiagen, Germantown, MD, USA) followed by passage through a Micro Bio-Spin P30 column (Bio Rad, Hercules, CA, USA), concentrated via ethanol precipitation, and resuspended in PCR-grade water at a final concentration of 0.5 μg/μL prior to electroporation. The plasmid used in the experiment shown in Figure 3 (#819) contains the entire 125 bp intergenic region upstream of the *P. polycephalum* mitochondrial *LSU* gene and 15 bp of the transcribed portion of the *LSU* gene inserted into the *Hind*III and *Sal*I sites of pBSM13+. The *LSU* gene was omitted from the dot blot to avoid potential hybridization to *LSU*-vector fusion transcripts. The plasmid used in the experiment shown in Figure 4 (#802) contains 70 bp of the intergenic region immediately upstream of the *P. polycephalum* mitochondrial *LSU* gene and 97 bp of the transcribed portion of the *LSU* gene inserted into the *Pst*I and *Xba*I sites of pBSM13+.

### 2.2. Mitochondrial Isolation

Although *P. polycephalum* mitochondria isolated via differential centrifugation synthesize run-on transcripts that are fully edited, when further purified on Percoll (GE Healthcare Life Sciences, Pittsburgh, PA, USA) gradients, few sediment at the appropriate density, resulting in very low yields. To enhance mitochondrial yields, we explored a broad range of conditions, ultimately arriving at the conditions described below. The mitochondria in these preparations are uniform in size and essentially free of nuclei, pigment granules, and other contaminants (Figure 1, left). They are also transcriptionally active and synthesize edited RNAs (see Results). 

Microplasmodia were cultured at 26 °C in 300 mL SDM medium [32] in a 2-L baffled flask to mid-log phase, then poured into a large beaker on ice and allowed to settle by gravity. The medium was decanted and the cells washed by swirling gently in 300 mL ice-cold sclerotia salts (19 mM citric acid, 0.3 mM FeCl_2_, 2.4 mM MgSO_4_, 8.1 mM CaCl_2_, 0.4 mM MnCl_2_, 0.1 mM ZnSO_4_, 2.9 mM KH_2_PO_4_, pH 4.8) [33]. The liquid was again decanted and the cells were resuspended in 75 mL ice-cold sclerotia salts and split evenly into two 50 mL conical tubes. Cells were pelleted by centrifugation for 2 min at ~800× *g* and the liquid was again decanted. Each pellet was washed twice with sterile ice cold distilled water, vortexing gently and pelleting as above. After removing as much liquid as possible, cells were transferred to a 55 mL Wheaton homogenizer (Millville, NJ, USA) on ice, rinsing the tubes with 15 mL of ice cold buffered sucrose (BSS, 10 mM Tris (pH 7.5)/0.25 M sucrose). Cells were broken open by douncing for 5 strokes and α-amylase was added to the lysate at a final concentration of 40–80 μg/mL. After 10 min on ice, cells were dounced another 5 strokes and the homogenate was examined using phase contrast microscopy to determine if additional strokes were needed. The lysate was then filtered to remove residual clumps and slime prior to layering over Percoll gradients consisting of steps of 1.082, 1.062, and 1.044 g/mL in 2 mM Tris (pH 7.5)/0.25 M sucrose. Gradients were centrifuged for 1 min at 25,000× *g* and the mitochondrial bands were transferred to a fresh tube. Mitochondria were then diluted by slowly layering 2.5 volumes of 2 mM Tris (pH 7.5)/0.25 M sucrose with gentle mixing prior to centrifugation at 7600× *g* for 10 min at 4 °C. Mitochondria were resuspended in 0.33 M sucrose and the protein content was determined (Bio-Rad Protein Assay) prior to pelleting at 5100× *g* for 5 min at 4 °C. The final mitochondrial pellets were resuspended in 0.33 M sucrose and kept on ice until use.

### 2.3. Electroporation

Mitochondria at protein concentrations of 5–10 μg/μL were split into 50 μL aliquots and mixed with plasmid DNA (0.5–1 μg) or purified PCR fragment (30–500 ng) as appropriate. Mitochondria were then transferred to a cold 0.1 cm gap electroporation cuvette (Bio Rad) and electroporated using an Eppendorf Electroporator 2510 (Hauppauge, NY, USA) at 10 μF, 600 Ω, and field strengths between 5 kV/cm and 20 kV/cm. The samples were immediately transferred to a fresh tube and the cuvette was rinsed with 50 μL 0.33 M sucrose, which was added to the sample. Samples were then used for microscopy and/or transcription and editing assays as noted.

### 2.4. Microscopy

Mitochondria were concentrated by pelleting and resuspended in 0.33 M sucrose prior to microscopy. Images were collected with a Retiga EXI camera (Q-imaging Vancouver, BC, Canada) mounted on a Leica DMI 6000 B inverted microscope (Leica Biosystems, Wetzlar, Germany) using a 63× objective (1.4 NA Plan Apo) with a 1.6× mag changer for a resulting magnification of 1008×. Mitochondria were visualized using phase contrast optics. Fluorescent DNA was visualized using a standard Texas Red filter set. Equal thresholds were set to detect only the brightest fluorescent signals. These signals were then outlined and overlaid onto the phase image. The co-localization of fluorescence and mitochondria is indicated by colored outlines in the overlay image. 

### 2.5. Transcription, RT-PCR, and PCR

Transcription reactions with unlabeled nucleotides were carried out in 0.3–0.33 M sucrose/20 mM Tris (pH 7.5)/20 mM MgCl_2_/10 mM KCl/2 mM DTT/500 μM NTPs. Mitochondria were pelleted and resuspended in Trizol Reagent (Invitrogen/Thermo Fisher Scientific, Waltham, MA, USA) and RNAs were isolated as specified by the supplier. The resulting RNA pellets were resuspended in 10 mM Tris (pH 7.5)/1 mM EDTA, extracted with 24:1 chloroform: isoamyl alcohol (CIA), and ethanol precipitated. To avoid background signal from residual exogenous DNA in subsequent PCR reactions, RNA preparations were subsequently incubated with *Eco*RI (New England Biolabs, Ipswich, MA, USA) to remove the primer binding sites for the reverse transcription and PCR reactions, CIA extracted, and ethanol precipitated prior to two rounds of DNase I (Roche, Indianapolis, IN, USA) treatment. Parallel complementary DNA (cDNA) synthesis reactions were carried out by annealing a primer complementary to sequences specific for the exogenous DNA (#508: GGTTTTCCCAGTCACGAC), then splitting each annealed RNA/primer mix into two equal aliquots, incubating under reaction conditions that were identical except for the presence or absence of AMV reverse transcriptase (Life Sciences, Saint Petersburg, FL, USA). Subsequent PCR reactions were carried out with primers #494 (TGTAAAACGACGGCCAGTG) and #505 (CTAGATCTGGGTCGTTGTC), yielding a 538 bp fragment (Figure 2). No signal was observed in the absence of added DNA, indicating that endogenous RNA does not yield a product with these primers (Figure 2B). The 847 bp PCR product used for electroporation (Figure 1 and Figure 2A) was generated from plasmid #809 using primers #508 (GGTTTTCCCAGTCACGAC) and #509 (5′-cy3–GAAACAGCTATGACCATG). PCR fragments for dot blots were generated using the primers listed in Table S1. All PCR products were generated with Taq DNA polymerase (New England Biolabs) under conditions specified by the supplier. 

### 2.6. Dot Blot Experiments

PCR fragments to be immobilized on nitrocellulose filters were heated to 95 °C for 5 min, snap-cooled on ice, then incubated in 0.5 M NaOH for 10 min on ice. Samples were neutralized by the addition of one-half volume of neutralization solution [0.5 M Tris-HCl (pH8)/217 mM Na citrate/1 M NaCl/1 M HCl] and 20 μL aliquots of denatured DNAs were spotted onto nitrocellulose filters using a vacuum manifold. Filters were air-dried, washed twice with 6X SSC (0.9 M NaCl/90 mM Na citrate), air-dried, UV cross-linked at 254 nm for 3 min, and baked for 2 hours at 80 °C. 

Mitochondria at a protein concentration of 8.25 μg/μL were split into three 50 μL aliquots; one sample was kept on ice, while the remaining aliquots were electroporated either in the absence or presence of plasmid #819 at a field strength of 10 kV/cm as noted. Transcription reactions were carried out at 30 °C in 0.33 M sucrose/20 mM Tris (pH 7.5)/20 mM MgCl_2_/10 mM KCl/2 mM DTT/250 μM ATP, CTP, and UTP/ 2 μM α^32^P-GTP for 15 min, then chased for 5 min with cold GTP as previously described [19]. Mitochondria were pelleted and total RNA was isolated with Zymo Spin IIC columns under conditions specified by the supplier to remove unincorporated nucleotides. After a 3 h pre-incubation of dot blot filters in hybridization buffer (0.5 M sodium phosphate (pH 7.2)/1% BSA/15% formamide/7% SDS/I mM EDTA), dot blots were incubated overnight with a fresh solution of hybridization buffer containing 4 × 10^5^ cpm labeled RNA per blot. Filters were washed once with 5X SSC/0.1% SDS, and twice with 2X SSC/0.1% SDS prior to visualization using a Typhoon phosphorimager (GE Healthcare, Life Sciences, Pittsburgh, PA, USA). Relative signal intensities were measured using ImageQuant software (Molecular Dynamics version 5.2, GE Healthcare, Life Sciences, Pittsburgh, PA, USA).

### 2.7. RNA Editing Assay

The presence of fully edited mitochondrial transcripts precludes assaying editing of newly synthesized RNA from endogenous genes via bulk RNA analysis, necessitating the use of labeling strategies. Transcripts were labeled under the same conditions used for the dot blot experiments, mitochondria were pelleted and total RNA was isolated with Zymo Spin IIC (Zymo Research, Irvine, CA, USA) columns. S1 nuclease protection and RNase T1 digestions were carried out as described by [34], using single stranded DNA complementary to the 3′ portion of the *atpA* mRNA as described in [17]. 

## 3. Results 

### 3.1. Efficient DNA Uptake upon Electroporation of Isolated P. polycephalum Mitochondria

Electroporation methods require highly purified mitochondria in relatively high concentration. In order to isolate mitochondria in sufficient quantities, we first developed a new mitochondrial purification method (see Materials and Methods). To generate DNA constructs that could support transcription within *P. polycephalum* mitochondria, the portion of the mitochondrial genome that spans the transcription start site of the highly expressed ribosomal RNA operon was cloned for use in electroporation experiments (see Materials and Methods). Plasmid DNA and PCR fragments derived from this fusion construct were used to assess DNA uptake in optimization experiments.

To optimize electroporation conditions for *P. polycephalum* mitochondria, the efficiency of DNA uptake at different field strengths was assessed using a 847 bp PCR fragment synthesized using a cy3-labeled DNA oligonucleotide. Uptake of the cy3-labeled PCR product by isolated *P. polycephalum* mitochondria was observed between 5 and 13 kV/cm, with 10 kV/cm being optimal. In the experiment shown in Figure 1, the purified cy3-labeled DNA fragment was mixed with a suspension of isolated mitochondria and split into three tubes. One third of the mixture was not subjected to electroporation, one third was electroporated at 10 kV/cm, and the final third was electroporated at 13 kV/cm. Mitochondria were then washed to remove cy3-labeled DNA that was not taken up by the mitochondria prior to fluorescence microscopy. As can be seen in the center panel of Figure 1, background levels of fluorescence were extremely low in mitochondria that were not subjected to electroporation (<5%). In contrast, efficient uptake of labeled DNA was observed upon electroporation at both 10 kV/cm (~85%) and 13 kV/cm (~85%) (Figure 1, center). Some spots are clearly brighter than others, which could either be a function of depth of field or, potentially, the result of a greater amount of DNA being taken up in these mitochondria. Although only the brightest spots show up well on the overlay (Figure 1 right), virtually the entire fluorescent signal co-localizes with mitochondria (compare phase and Texas Red patterns in Figure 1). DNA uptake was ~4–5 fold lower at 5 kV/cm and 7.5 kV/cm, while at field strengths above 12 kV/cm the number of intact mitochondria was significantly reduced (e.g., 13 kV/cm in Figure 1). These results demonstrate that DNA uptake under these conditions is highly efficient and is dependent upon electroporation. 

### 3.2. DNA Introduced into Mitochondria is Transcribed

To determine whether the DNA taken up by *P. polycephalum* mitochondria is transcribed by the mitochondrial RNA polymerase, a plasmid containing the *LSU-cox1* fusion construct or the 847 bp cy3-labeled PCR fragment derived from this construct was used for electroporation. Mitochondria were recovered, incubated under conditions that support robust RNA synthesis [35], and total mitochondrial RNA was isolated. cDNA synthesis and subsequent PCR was carried out using primers specific for the fusion construct to avoid amplifying endogenous transcripts. RT-PCR products of the expected size were produced from RNAs isolated from mitochondria electroporated in the presence of the PCR product (Figure 2A, lanes 2 and 4) or plasmid DNA (Figure 2B, lane 4). No signal was observed when reverse transcriptase was omitted from the cDNA synthesis reactions (Figure 2A,B, lanes 3, 5, 7), when DNA was omitted (Figure 2B, lane 6), or in the absence of electroporation (Figure 2A, lane 6 and Figure 2B, lane 2). RT-dependent PCR products of the correct size were observed over a range of DNA concentrations tested (30–500 ng of the PCR fragment, 0.5–1 μg of a 3.9 kbp plasmid DNA). These results demonstrate that DNA taken up upon electroporation is expressed as RNA.

### 3.3. Transcripts Derived from DNA Introduced into Mitochondria Are Not Edited

The constructs used in these experiments contain a total of 21 editing sites, including the first editing site within the large rRNA (*LSU*) and 20 editing sites within the *cox1* region, which includes both insertion sites (13 +C, 1 +U, 1 +UA, and 1 +CU sites) and 4 sites of C to U changes. This allowed us to examine the extent of editing at multiple types of editing sites. Use of an *LSU*-specific primer in the PCR reactions led to spurious bands arising from the endogenous rRNA, so RNA editing was only assessed at the 20 *cox1* editing sites. To determine whether the RNAs produced from DNA introduced into mitochondria were edited, bulk RT-PCR products derived from these transcripts were sequenced. No evidence of editing at any of the sites within the *cox1* portion of the fusion transcript was observed under any of the conditions tested thus far (representative example shown in Figure S1). These include the conditions used in various plant mitochondrial electroporation experiments [23,29,36] and conditions that support extensive RNA editing in *P. polycephalum* mitochondria [19,35,37,38] (Figure S1).

### 3.4. Electroporation Does Not Appreciatively Alter the Level of Transcription from Endogenous Genes

Based on the lack of editing from exogenous templates, we next tested whether electroporation had a deleterious effect on *endogenous* gene expression. The level of transcription of 18 of the 20 transcripts identified in our analysis of the mitochondrial transcriptome (Figure 3A) was assessed. Newly synthesized transcripts were labeled to distinguish them from pre-existing RNA pools and used to probe dot blots loaded according to the grid shown in Figure 3B. In this series of experiments, mitochondria were isolated from microplasmodia and the suspension was divided into thirds. Run-on transcription was monitored by incorporation of α^32^P-GTP into RNA in mitochondria not subjected to electroporation and mitochondria that had been electroporated in the absence or presence of exogenous DNA. To assess transcription levels relative to expression of endogenous genes, this plasmid (#819) contained the mitochondrial *LSU* promoter driving expression of vector sequences. Total RNA was isolated and labeled RNA from each sample was used to probe individual dot blots on which PCR products from 19 mitochondrial genes, three nuclear genes, and the plasmid vector were immobilized. The relative level of gene expression was similar between the three samples (Figure 3C,D). Transcripts from all 19 mitochondrial genes were detected, with expression levels between genes varying over a 60-fold range, while spots corresponding to nuclear genes gave only background signal (Figure 3C,D). Upon prolonged exposure, an extremely low level of transcription from the plasmid sequences downstream of the *LSU* promoter region was detected. Transcription from the introduced plasmid DNA was ~7-fold lower than the level of the weakest signal from an endogenous mitochondrial gene and was only present in mitochondria that had been electroporated in the presence of exogenous DNA, as expected. This is not surprising given that the signal from endogenous genes is primarily derived from run-on transcription of previously initiated transcripts, whereas the signal corresponding to vector sequences (pBSM13+) is solely due to newly initiated transcription from the LSU promoter inserted into the plasmid. These experiments indicate that electroporation does not significantly affect transcription from the mitochondrial genome.

### 3.5. Insertional Editing Occurs within Transcripts from Endogenous Genes after Electroporation

One potential explanation for the lack of insertion editing within transcripts synthesized from exogenous DNA was that electroporation disrupts editing in some way. To determine whether the editing apparatus is still functional after electroporation, editing was assessed in the pool of labeled run-on transcripts synthesized under the same conditions as those used for the dot blot analysis shown in Figure 3. To isolate transcripts from the endogenous *atpA* gene, total RNA from each sample was hybridized to single-stranded DNA complementary to a portion of the *atpA* mRNA, then digested with S1 nuclease. The protected region of the *atpA* mRNA was gel purified, digested with RNase T1, and the migration of the resulting fragments was compared to those from S1-protected unedited and edited control transcripts. As observed previously, transcripts made in isolated mitochondria prior to electroporation (Figure 4, lane 3) are fully edited, i.e., the migration of the diagnostic RNase T1 fragments indicated on the left matches those in the edited control (lane 2). Editing sites 52 (16/17mer) and 53 (20/21mer) are also fully edited in mitochondria after electroporation in either the absence (M+, lanes 4 and 9) or presence (M + D, lanes 5 and 10) of plasmid DNA. Editing efficiency was reduced at sites 50 (29/30mer, ~50% edited) and 54 (24/25mer, ~85% edited) in the electroporated samples (lanes 4, 5, 9, 10), indicating that electroporation does have some effect on the editing machinery, at least at a subset of sites. However, substantial levels of editing are observed at all sites, demonstrating that mitochondria remain editing competent after electroporation. Thus, the lack of editing from exogenously supplied DNA cannot be simply due to global inactivation of the editing machinery. 

## 4. Discussion

Run-on transcripts synthesized in *P. polycephalum* mitochondria isolated via differential centrifugation with or without subsequent gradient purification are completely edited at most insertion sites under a range of transcription conditions [19,35,37,38]. However, mitochondrial yields are quite low upon secondary purification on Percoll gradients. In order to obtain sufficient quantities of mitochondria for electroporation experiments, a new purification scheme was developed. This involved the use of gentler lysis conditions, direct layering of filtered lysates onto Percoll step gradients, and minimization of applied centrifugal force (see Materials and Methods). Mitochondria purified in this way are free of contaminating nuclei, membranes, and pigment granules (Figure 1), are capable of taking up both linear and circular DNAs (Figure 1 and Figure 2), express the normal complement of endogenous genes (Figure 3), and synthesize edited transcripts (Figure 4). 

Electroporation methods have been developed for plant mitochondria from both monocots and dicots. Plasmid constructs introduced into mitochondria isolated from wheat embryos [23], etiolated maize seedlings [29], and potato tubers [31] support transcription, splicing, and C to U editing, providing a means of investigating editing signals [22,25,26] and the interplay between different forms of RNA processing [27,29,30,31]. The studies in plant mitochondria utilized two distinct sets of electroporation and expression conditions, and variations of each were tested using isolated *P. polycephalum* mitochondria. None of these conditions supported editing of transcripts derived from DNA constructs introduced into *P. polycephalum* mitochondria. Switching to conditions known to support insertion editing in *P. polycephalum* mitochondria also failed to result in editing of transcripts from exogenous genes (Figure S1), despite the fact that native transcripts were largely edited under the same conditions (Figure 4). Curiously, although transcription from the mitochondrial genome was unaffected (Figure 3), electroporation reduced the level of editing at some sites but not others within the endogenous transcripts, although all sites examined were edited to at least 50%. The reason for this is not clear, given that all sites were fully edited in untreated mitochondria processed in parallel (Figure 4). The finding that cells electroporated in the absence or presence of DNA both yielded the same editing pattern, with some sites being fully edited, argues against the possibility that editing is only affected in the ~85% of mitochondria that have taken up DNA.

The inability of DNA introduced into isolated *P. polycephalum* mitochondria to support editing may be due to the lack of associated editing factors. We have previously demonstrated that insertion editing requires a factor or factors associated with the *P. polycephalum* mitochondrial genome [39]. Fractionation of mitochondrial lysates over a gel filtration column yields crude mitochondrial transcription elongation complexes (mtTECs) that are editing competent [18]. The DNA in these complexes can be digested with restriction enzymes and the resulting fragments can be ligated to create chimeric templates. RNAs produced from intra- and inter-genic fusions are edited to the same extent as RNAs produced by mtTECs. However, when exogenous DNA fragments are ligated to digested mtTEC fragments, the portion of the run-on RNAs produced from the native template is edited, but the portion derived from the added DNA is not. Importantly, the same is true when deproteinized fragments of *P. polycephalum* mitochondrial DNA are fused to mtTEC templates, strongly suggesting a role for a DNA-bound factor(s) [39]. 

The lack of editing in transcripts derived from exogenous templates is unlikely to be due to context effects based on our previous work with chimeric templates, which utilized dozens of intra- and intergenic chimeras, including rRNA–rRNA, mRNA–mRNA, and rRNA–mRNA fusions [20,39,40]. Using chimeric templates with junctions very close to editing sites, we demonstrated that the *cis*-acting elements required for the insertion of non-encoded nucleotides are limited to template sequences within 9 bp upstream and 9–10 bp downstream of an editing site [20]. Template changes outside of this critical region had no effect on the level of editing at any site within the chimeric RNAs, many of which contained sequences derived from the *LSU* gene. Thus, even if inclusion of *LSU* sequences upstream of the *cox1* region resulted in inhibitory RNA structures, there is no reason to believe that all 20 of the downstream editing sites would be negatively affected. 

The work presented here was initiated to determine whether a lack of editing factors could be overcome by expressing exogenous DNA templates within the confines of isolated mitochondria. The finding that editing was not observed when either linear or circular DNA was introduced into mitochondria and transcribed under a variety of conditions may be due to the absence of some component needed for de novo assembly of editing complexes. Additional conditions, such as inclusion of amino acids to support new protein synthesis, will be explored in future studies.

## Supplementary Materials

The following supplementary materials can be found online at www.mdpi.com/2073-4425/7/12/128/s1, Figure S1: Transcripts from DNA introduced into mitochondria are not edited. Sequence trace of the bulk RT-PCR product shown in lane 4 of Figure 2B (shown again at the top of this figure). The positions of *cox1* editing sites (es) 22–43 and the 5 bp block mutation (block mt) are indicated on the trace. Black arrowheads represent sites where nucleotides are normally added to mitochondrial transcripts (including 1 single U insertion site, 13 single C insertion sites, and the UA and UC insertion sites); blue arrowheads represent the 4 sites of expected C to U changes. No editing is observed at any of these sites, Table S1: Oligonucleotides used to generate PCR fragments immobilized on dot blot filters.
