# Supplementary material for: Electroporation of DNA into Physarum polycephalum Mitochondria: Effects on Transcription and RNA Editing in Isolated Organelles
Source: Genes (Basel). 2016 Dec 14;7(12):128. doi: 10.3390/genes7120128 (PMC5192504; doi:10.3390/genes7120128)
Supplement: Supplementary file 1 [file genes-07-00128-s001.docx]

Supplementary Materials: Electroporation of DNA into *Physarum polycephalum* Mitochondria:
Effects on Transcription and RNA Editing in
Isolated Organelles

Jonatha M. Gott, Gregory M. Naegele and Scott J. Howell


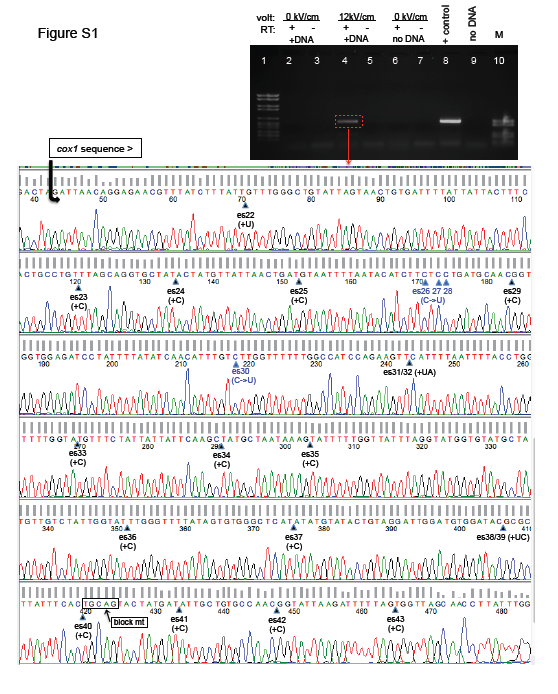


**Figure S1.** Transcripts from DNA introduced into mitochondria are not edited. Sequence trace of the bulk RT-PCR product shown in lane 4 of Figure 2B (shown again at the top of this figure). The positions of *cox1* editing sites (es) 22-43 and the 5 bp block mutation (block mt) are indicated on the trace. Black arrowheads represent sites where nucleotides are normally added to mitochondrial transcripts (including 1 single U insertion site, 13 single C insertion sites, and the UA and UC insertion sites); blue arrowheads represent the 4 sites of expected C to U changes. No editing is observed at any of these sites.

**Table S1.** Oligonucleotides used for generating PCR products for dot blots.

| **Gene** | **oligo ID#** | **Oligonucleotide Sequence** |
| --- | --- | --- |
| *nad5* | #31 | 5′-CAGCTACTGATCAAGATTC-3′ |
|  | #394 | 5′-AAAGTCGACTCCGAGGACAAAGATTATG-3′ |
| *rpS2* | #415 | 5′-AAGGATCCGAGTAACAGAGCTATTAGAG-3′ |
|  | #416 | 5′-AAAGTCGACTAGTATTAGATACTTCAGC-3′ |
| *rpS12* | #395 | 5′-AAGGATCCAACCTAATTCTGCAAACGCA-3′ |
|  | #396 | 5′-AAAGTCGACCACACCATATTTACTACAC-3′ |
| *php15* | #397 | 5′-AAGGATCCGAATTATAGGCCAGAC-3′ |
|  | #398 | 5′-AAAGTCGACTCATAATACTGAGGATGG-3′ |
| *coI* | #36 | 5′-AAGGATTATAAACAAATTCCC-3′ |
|  | #44 | 5′-GGTATGGTGTATGCTATG-3′ |
| *co2* | #7 | 5′-GATTAACTCCACATAATTC-3′ |
|  | #6 | 5′-GGACATTAATTCCTTCTTT-3′ |
| *nad2* | #322 | 5′-GTTTTATTAGCAGTATTGGG-3′ |
|  | #318 | 5′-TAAAGCAAAGACAGTACCTA-3′ |
| *atp8/nad4L* | #295 | 5′-CAGCTTCTAATAAAAGCTAAC-3′ |
|  | #294 | 5′-GGTTATGATCATAAGAGCAAA-3′ |
| *nad4* | #346 | 5′-CAAAATATTGGCATAACAGTAAC-3′ |
|  | #74 | 5′-TTTATGGCTTCCTAAAGCC-3′ |
| *nad3* | #338 | 5′-AGAAACTAACAATATGGCGAG-3′ |
|  | #336 | 5′-GTCAATTTGATAAAAAGTAGTTG-3′ |
| *nad9* | #469 | 5′-AAAGCCGTAGATCTAGACA-3′ |
|  | #470 | 5′-TCGACCTTTATTTTTGTACG-3′ |
| *tRNA/EM1* | #114 | 5′-CCTGGTGCCCCTTGAGAGAATCG-3′ |
|  | #195 | 5′-AAACGTGTGCCTCATGA-3′ |
| *SSU* | #206 | 5′-ATGGCGTGAGCCTGAGCA-3′ |
|  | #187 | 5′-AGCCCTGTATGCGAACC-3′ |
| *atpA* | #96 | 5′-CTTAAACAAGGTCCTAATATG-3′ |
|  | #97 | 5′-AGAATTCCGGTTCAAAACTT-3′ |
| *nad6* | #299 | 5′-TCTAGTAATTTATTAGAAAACCA-3′ |
|  | #301 | 5′-CATAAGCGTGTATGAGCG-3′ |
| *rpL16* | #487 | 5′-AACGATGTACTTTACGTAGT-3′ |
|  | #492 | 5′-TTAGAGACTGCACGTAGAG-3′ |
| *nad1* | #411 | 5′-AAGGATCCGAGCATTATGTTGCTCTCC-3′ |
|  | #412 | 5′-AAAGTCGACGAATAGTTGTGTAAACGACC-3′ |
| *cytb* | #131 | 5′-GTAGCTGCCCAATAAGACAT-3′ |
|  | #203 | 5′-TGATACAGCCATAACTTATC-3′ |
| *atp9* | #211 | 5′-TTAAAGAAGATTTTGTAAATTC-3′ |
|  | #212 | 5′-AAAGCCATCATCAAAGCC-3′ |
| *Tubulin* | #82 | 5′-TCGCTCAGCTATGGGATTC-3′ |
|  | #81 | 5′-CTCGACAGCGTCGTTCTG-3′ |
| *pBSM13+* | #499 | 5′-CGGAATTAACCCTCACTAAAG-3′ |
|  | #544 | 5′-AAAGGAAGGGAAGAAAGCGAA-3′ |
| *RNAP* | #475 | 5′-GAGGACAAGCAGGCATTC-3′ |
|  | #157 | 5′-ACGTGGCCGTCAACAAC-3′ |
| *glom2* | #540 | 5′-CCTGCGTAGCTCACAGAAG-3′ |
|  | #543 | 5′-CCTCAGACACGAATCTCC-3′ |
